# Coeliac disease and C virus-related chronic hepatitis: a non association

**DOI:** 10.1186/1756-0500-5-533

**Published:** 2012-09-26

**Authors:** Antonietta Gerarda Gravina, Alessandro Federico, Mario Masarone, Antonio Cuomo, Concetta Tuccillo, Carmelina Loguercio, Marcello Persico, Marco Romano

**Affiliations:** 1 Department of Clinical and Experimental Medicine and Surgery “F Magrassi e A Lanzara” – Hepato-Gastroenterology Division and C.I.R.A.N.A.D., Second University of Naples, via Pansini 5, Naples 80131, Italy; 2 Internal Medicine and Hepatology Unit, Second University of Naples, via Pansini 5, Naples, 80131, Italy; 3 Gastroenterology Unit, Umberto I Hospital, Nocera Inferiore, Italy

**Keywords:** Coeliac disease, HCV-related chronic hepatitis, Liver, α-interferon therapy

## Abstract

**Background:**

A higher prevalence of coeliac disease has recently been reported among patients with HCV-related chronic hepatitis. Moreover, development of clinically overt coeliac disease has been described in a number of HCV-related chronic hepatitis patients during α-interferon therapy. This prospective study was designed to evaluate 1) the prevalence of coeliac disease in patients with HCV-related chronic hepatitis; 2) the prevalence of HCV infection in patients with coeliac disease; 3) whether PEG interferon-α treatment might favour the development of coeliac disease in patients with chronic hepatitis C.

**Materials and methods:**

Two hundred-ten consecutive patients (M/F = 140/70, range of age 35–58 years, median age 46.5 years) with biopsy proven chronic hepatitis C underwent serological screening for antiendomysial and tissue transglutaminase IgA antibodies. One hundred ninety-four coeliac patients (M/F = 52/142, range of age 18–74 years, median age 34 years) were screened for HCV antibodies. Positivity for HCV antibodies in coeliac disease patients was confirmed by detection of serum HCV-RNA by RT-PCR. This work was carried out in accordance to ethical guidelines of Declaration of Helsinki and was approved by Institutional Ethics Committee of the Second University of Naples. All patients gave informed written consent.

**Results:**

1) none of the 210 HCV-related chronic hepatitis patients were positive for coeliac disease serologic screening; 2) prevalence of HCV infection among coeliac patients was 1.54% (3/194) which is comparable to that reported in the Southern Italy population; 3) PEG interferon-α treatment was not associated with development of coeliac disease either clinical or serological.

**Conclusions:**

1) coeliac disease is not associated with HCV infection; 2) PEG interferon-α does not trigger celiac disease.

## Background

Coeliac disease (CD) is a condition characterized by damage to the mucosa of the small bowel in sensitive individuals and subsequent malabsorption, with a prevalence in the Western Countries of about 1/100 [1-3]. The clinical spectrum ranges from overt malabsorption to no clinical signs when the damage of the small intestine mucosa is mild (i.e. latent CD) [1]. CD is the only human autoimmune disease in which an environmental factor (i.e., gluten) has been identified that triggers an immune-mediated injury to the intestine in genetically predisposed individuals [4,5].

Hepatitis C virus (HCV) infection is the major cause of chronic liver disease worldwide [6]. Moreover, HCV infection is associated with a number of autoimmune disorders such as cryoglobulinemia, Sjogren syndrome, and lichen planus [7,8].

Even though the association between CD and liver diseases has been extensively investigated, a definite correlation between these pathological conditions has not been unequivocally established. An increased prevalence of CD has been described by Fine *et al.* in patients with HCV-related chronic hepatitis, thus leading to speculate that CD is epidemiologically associated with HCV-related chronic hepatitis [9]. In this study, four patients tested positive for specific markers of CD among 259 patients infected with HCV [9]. In another study, one patient with CD was found to have HCV infection when investigators looked for causes of increased alanine aminotransferases (ALT) levels in this setting [10]. Also, liver involvement is a frequent finding in CD patients [11]. On the other hand, Germenis *et al.* found a 0.54% prevalence of positive CD serology among 738 patients with liver disease, which was not different than what found in the general population [12]. Therefore, whether CD is part of the spectrum of HCV infection-related autoimmune disorders is still controversial [13].

A number of cases of clinically overt CD have been described in patients with HCV-related chronic hepatitis during treatment with interferon alpha (IFN-α) [14-17]. Also, Hernandez *et al.*[18] suggested that IFN-α may precipitate the development of CD in susceptible individuals. On the other hand Ruggeri *et al.* failed to demonstrate development of positive CD serology in 42 HCV-infected patients treated with IFN-α. Therefore, whether IFN-α is able to trigger CD is still unclear.

In order to specifically address these issues we designed a prospective study aimed at evaluating the prevalence of CD in patients with HCV-related chronic hepatitis and the prevalence of HCV infection in a population of patients with CD. Moreover, we studied whether pegylated (PEG) IFN-α treatment might be associated with the development of CD.

## Methods

### HCV-related chronic hepatitis patients

The study population consisted of 210 consecutive patients (M/F = 140/70, range of age 35–58 years, median age 46.5) with a biopsy proven HCV-related chronic hepatitis enrolled from September 2008 to July 2010. All these patients were tested for routine liver function tests (i.e., aminotranferases, γ-glutamyltranspeptidase, alkaline phosphatase, bilirubin, prothrombin activity, cholinesterase), immunoglobulins (Ig)A, IgG, and IgM, platelet count, blood cell count, haemoglobin, albumin, HBsAg, HBsAb, HBcAb (IgM-IgG), HBeAg, HBeAb, HCVAb, ANA, AMA, SMA, anti-LKM, plasma iron and copper levels, ceruloplasmin and ferritin and for antibodies against endomysium (EMA) tested on thin sections of human cord using an indirect immunofluorescent method and for tissue transglutaminase (tTG) by using ELISA assays. All underwent ultrasonography.

One hundred and sixty eight patients with HCV-related chronic hepatitis naïve to treatment (M/F = 125/43, range of age 35–52 years, median age 44) were eligible for interferon therapy and were treated with standard of care (PEG interferon-α plus ribavirin). These patients underwent screening for coeliac disease before the treatment and at 24 and 48 week of treatment.

This work was carried out in accordance to ethical guidelines of Declaration of Helsinki and was approved by Institutional Ethics Committee of the Second University of Naples. All patients gave informed written consent.

### RNA preparation and HCV-RNA determination

All steps were carried out under RNase-free conditions. The polymerase chain reaction (PCR) procedure was used to determine HCV RNA. Sera were rapidly (within 30 min of blood drawing) frozen at −20°C. RNA was extracted according to Chomczynsky and Sacchi [19], and c-DNA was derived. To identify HCV-RNA, a nested PCR was performed using primers that expanded the highly conserved 5’ non-coding genomic region. Carryover PCR contamination was avoided by applying the measures suggested by Kwok and Higuchi [20]. Limit of the house virus detection test applied was of 10 copies/ml.

### HCV genotyping

The genotype analysis of HCV was performed using a commercial hybridization assay (Inno Lipa HCV II; Innogenetics, Gent, Belgium); utilizing HCV-positive amplification products from the PCR assay (Amplicor HCV Amplification 2.0; Roche Diagnostics, Indianapolis, IN, USA). Serum PCR products were hybridized to type- and subtype-specific probes 1a, 1b, 2a, 2b and 3a, in order to classify the HCV genotypes. The probes used were to fulfill two main criteria: no more than two mismatches to three corresponding published sequences of the same subtype and they were to differ by three or more mismatches compared to published sequences of other types and subtypes. The only exception is probe 2b showing only two mismatches to the corresponding sequence of type 3a [21].

#### Liver biopsy and histology

Hepatic percutaneous biopsy was performed with a Surecut 17G needle via the intercostal route and an echo-assisted method. Liver specimens were used for histological examination if they were at least 1.5-cm long and contained ≥ 5 portal spaces. Specimens were fixed in formalin, embedded in paraffin, and stained with hematoxylin-eosin, Red Sirius, Ubiquitin, trichrome, and Prussian blue. Steatosis was graded 1 (<33% of hepatocytes), 2 (33-66% of hepatocytes), 3 (>66% of hepatocytes). Biopsies were evaluated with the Ishak score [22], and biopsies with steatosis were also scored according to Brunt’s criteria [23]. Table 1 includes the main clinical and laboratory characteristics of patients with HCV-related chronic hepatitis.

**Table 1:** Main clinical, biochemical and histological features of the 210 patients with HCV-related chronic hepatitis

	**N = 210**
Male/Female	140/70
Age (years; mean ± SD)	46.5 ± 11.56
Body Mass Index (mean ± SD)	26.7 ± 4.01
HCV-RNA levels (I.U./mL; mean ± SD)	4.318.618 ± 28.174
ALT (I.U./L) (mean ± SD) (n.v. < 40 U/I)	125 ± 101
HCV genotype	
1	168 (80%)
2	42 (20%)
Liver fibrosis (Ishak score)	
0	0
1	44.8%
2	33.7%
3	16.5%
4	5%

### Coeliac patients

We also studied 194 coeliac patients (M/F = 52/142, range of age 18–74 years, median age 34 years). The diagnostic workup included a thorough medical history, routine biochemistry, determination of serological markers of HBV and HCV infection. CD diagnosis was made on the basis of positivity to EMA and tTG of histological evaluation of duodenal biopsy samples showing subtotal/total villous atrophy, crypt hyperplasia and lympho-plasmacellular infiltration in the lamina propria [24]. Table 2 indicates the prevalence of associated autoimmune disorders in our coeliac patients. Coeliac patients who were positive for HCV antibodies were also tested for serum qualitative and quantitative HCV-RNA and HCV genotyping.

**Table 2:** Prevalence of autoimmune disorders in our series of 194 coeliac subjects

	**N**	**(%)**
Hashimoto thyroiditis	32	16.5
Dermatitis herpetiformis	2	1.0
Alopecia	1	0.5
Vitiligo	1	0.5
Primary biliary cirrhosis	1	0.5
Recurrent oral aphtosys	1	0.5
IgA mesangial nephropathy	1	0.5

#### Statistical analysis

Difference between groups was assessed by Fisher’s exact test. A value of p < 0.05 was considered statistically significant.

## Results

None of the 210 patients with HCV-related chronic liver disease resulted positive to serological screening for CD, nor did any of them show deficiency for IgA (Table 3). Furthermore, 3/194 (1.54%) CD patients were positive for both HCV antibodies and qualitative HCV-RNA in the serum (Table 3). This is comparable to the prevalence of HCV infection in Southern Italy [25]. One of the HCV positive CD patients had a history of intravenous heroine use, one had received blood transfusions for anemia before the diagnosis of CD was made and one was married to an HCV positive subject. Table 4 shows the main features and the degree of duodenal damage at histology of the HCV positive CD subjects.

**Table 3:** Prevalence of coeliac disease in HCV-related chronic hepatitis patients and prevalence of HCV-related chronic hepatitis in coeliac disease patients

	**HCV-related chronic hepatitis**	**Coeliac disease**
	**(n = 210)**	**(n = 194)**
Coeliac Disease	0/210	-
HCV-related Chronic Hepatitis	-	3/194

**Table 4:** Main characteristics of the three HCV positive coeliac patients

	**Patient 1**	**Patient 2**	**Patient 3**
Gender	male	female	female
Age	38	52	68
HCV genotype	2	1	1
Liver fibrosis score	2	2	1
Risk factors for HCV infection	History of i.v. heroin use	Married to subject with HCV infection	Blood transfusions
Duodenal histology degree	Marsh III a	Marsh III a	Marsh III b
Celiac disease symptoms at presentation	Syderopenic anaemia, weakness, bloating	Syderopenic anaemia	Chronic diarrhoea, weight loss, syderopenic anaemia

In order to assess whether IFN-α treatment might trigger CD we determined serum EMA and tTG in HCV-related chronic hepatitis patients before the treatment and at 24 and 48 week of treatment. One hundred and thirty patients completed the therapy, while thirty-eight discontinued for serious adverse events (54% anemia, 34% leukopenia, 12% thrombocytopenia). A virological (i.e. disappearance of serum HVCV-RNA) and biochemical (normal AST and ALT serum levels) was observed at the end of treatment in 91/130 (70%) of patients. None of the 130 patients who completed therapy turned positive for EMA or tTG nor did any of them develop clinical signs or symptoms suggestive for CD at any time point during the follow-up period.

## Discussion

Viral infections have been thought to act as a potential trigger for the development of CD in genetically susceptible individuals [26,27]. In particular, because of its association with a number of autoimmune disorders, the role of HCV infection in the pathogenesis of CD has been the object of several investigations, but the results are controversial [9-13].

In order to specifically address this issue we prospectively evaluated the prevalence of CD in 210 HCV-related chronic hepatitis patients. None of these patients had clinical signs or symptoms suggestive for CD nor did any of them show positivity for serological markers of CD (EMA and/or tTG). Moreover, in a population of 194 CD patients, we found a prevalence of HCV infection as low as 1.54%, which is not higher than the prevalence of HCV infection in Southern Italy [25]. Also, all of the CD patients who resulted HCV positive were at risk for HCV infection. This suggests that HCV-related chronic hepatitis and CD are not associated disorders. In partial support of our finding, Teml and Vogelsang in a retrospective study found that 3/488 (0.6%) CD patients were positive for HCV infection [13]. However, all of them were at risk for HCV infection, one patient having a history of i.v. drug abuse and two having received blood transfusion because of severe anaemia. Also, more recently, Thevenot *et al.* found 0% prevalence of CD in 624 consecutive patients with HCV infection [28]. Finally, our results are in agreement with the findings of Hernandez *et al.* who found no association between CD and HCV both in a retrospective and prospective analysis [18]. However, a report by Ruggeri *et al.* investigated CD-related antibodies in a large series of HCV-infected subjects that were also tested for non-organ-specific autoantibodies as surrogate marker of autoimmune disorders. The authors found that EMA occurred in 5/244 (2%) of HCV patients, which was significantly higher than the prevalence of EMA positivity found in healthy controls [29]. However, only in three of these patients CD was confirmed histologically and, in one of them, non-organ-specific autoantibodies were positive [29].

IFN-α treatment has been shown to potentially trigger autoimmune disorders [22]. Also, development of clinically overt CD has been described in patients with HCV-related chronic hepatitis during IFN-α treatment [14-17]. However, in these reports, half of the patients were EMA and tTG positive with no clinical or biochemical signs of malabsorption before starting IFN-α therapy whereas the EMA and tTG status was not available in the remaining patients. This suggests that IFN-α may trigger overt CD in asymptomatic EMA and tTG positive subjects (i.e. latent CD patients). However, the question as to whether IFN treatment may induce EMA and tTG positivity with/without malabsorption remains unanswered. We therefore prospectively studied whether EMA and tTG negative, chronic hepatitis C patients might develop clinically overt or latent CD during IFN-α plus ribavirin combined therapy. None of these patients showed clinical signs or symptoms suggestive for CD or positivity for EMA and tTG before or after treatment, thus making it unlikely that IFN-α may serve as a trigger for CD. This is also in agreement with Ruggeri *et al.* who found no CD-related antibody in 42 HCV-infected patients treated with IFN-α [29]. More recently, Hernandez *et al.* found that 6/878 CD patients had concomitant HCV infection and that in four of these 6 patients, symptoms that prompted CD diagnosis occurred during or after IFN-α therapy. This may suggest that IFN-α therapy may precipitate the development of CD in susceptible individuals. However, none of these subjects had been screened for CD prior to start IFN-α therapy [18].

A limit of this study is represented by the small number of patients studied. Also, this study lacked a control group of patients with chronic hepatitis other than HCV-related. However, we evaluated the prevalence of coeliac disease in a group of 131 patients with non alcoholic fatty liver disease (NAFLD) who were admitted to our GI Unit over the same period of time and found that none of these patients had a serology positive for coeliac disease (Romano M and Persico M unpublished observation). Similarly, the prevalence of HCV infection found in our coeliac patients (i.e. 1.5%) is comparable to that found in 315 non coeliac patients who underwent screening for HCV prior to esophagogastroduodenoscopy (i.e. 1.7%, p, ns) (Romano M unpublished observation).

## Conclusions

In summary, 1) none of HCV infected patients showed positivity for CD serology; 2) prevalence of HCV infection among CD patients was comparable to that of general population; 3) none of IFN-α-treated HCV patients developed CD. Based on this study, we conclude that HCV infection and CD do not seem to be epidemiologically associated. Moreover, IFN-α treatment seems unlikely to cause CD.

## Competing interests

The Authors declare no financial arrangements related to the research or assistance with manuscript preparation.

We did not receive and we will not receive reimbursements, fees, funding, or salary from any organization that may in any way gain or lose financially from the publication of this manuscript.

We do not hold any stocks or shares in an organization that may in any way gain or lose financially from the publication of this manuscript, either now or in the future.

We do not hold or we are not currently applying for any patents relating to the content of the manuscript. We have not received reimbursements, fees, funding, or salary from an organization that holds or has applied for patents relating to the content of the manuscript.

We have not any financial competing interests and we have not non-financial competing interests (political, personal, religious, ideological, academic, intellectual, commercial or any other) to declare in relation to this manuscript.

## Authors’ contributions

AGG and AF equally contributed to this manuscript. AGG, AF, MR have made substantial contributions to conception and design; AGG, AF, MR, AC, MM, CL, MP, CT have made analysis and interpretation of the data; AGG, AF, MR have been involved in drafting the manuscript and revising it critically for important intellectual content; MR has given final approval of the version to be published. All authors read and approved the final manuscript.

## References

[B1] Biagi F Corazza GR Clinical features of celiac disease Dig Liver Dis 2002 34 225 228 10.1016/S1590-8658(02)80197-9 11990396

[B2] Mäki M Mustalahti K Kokkonen J Kulmala P Haapalahti M Karttunen T Ilonen J Laurila K Dahlbom I Hansson T Höpfl P Knip M Prevalence of celiac disease among children in Finland N Engl J Med 2003 348 2517 2524 10.1056/NEJMoa021687 12815137

[B3] West J Logan RF Hill PG Lloyd A Lewis S Hubbard R Reader R Holmes GK Khaw KT Seroprevalence, correlates, and characteristics of undetected celiac disease in England Gut 2003 52 960 965 10.1136/gut.52.7.960 12801951 PMC1773707

[B4] Dieterich W Ehnis T Bauer M Donner P Volta U Riecken EO Schuppan D Identification of tissue transglutaminase as the autoantigen of celiac disease Nat Med 1997 3 797 801 10.1038/nm0797-797 9212111

[B5] Sollid LM Molecular basis of celiac disease Annu Rev Imm 2000 18 53 81 10.1146/annurev.immunol.18.1.53 10837052

[B6] Steinke DT Weston TL Morris AD MacDonald TM Dillon JF Epidemiology and economic burden of viral hepatitis: an observational population based study Gut 2002 50 100 105 10.1136/gut.50.1.100 11772975 PMC1773069

[B7] Zignego AL Brèchot C Extrahepatic manifestations of HCV infection: facts and controversies J Hepatol 1999 31 369 376 10.1016/S0168-8278(99)80239-6 10453955

[B8] Persico M De Marino FA Di Giacomo Russo G Persico E Morante A Palmentieri B Torella R Prevalence and incidence of cryoglobulins in hepatitis C virus-related chronic hepatitis patients: a prospectic study Am J Gastroenterol 2003 98 884 888 12738472 10.1111/j.1572-0241.2003.07339.x

[B9] Fine KD Ogunji F Saloum Y Beharry S Crippin J Weinstein J Celiac sprue: another autoimmune syndrome associated with hepatitis C Am J Gastroenterol 2001 96 138 145 10.1111/j.1572-0241.2001.03464.x 11197243

[B10] Múgica F Aranzadi MJ Recasens M Almagro F Muñagorri A Elósegui E Resano A Huarte I Adult celiac disease and hypertransaminasemia Rev Esp Enferm Dig 2000 92 78 85 10757865

[B11] Hagander B Berg NO Brandt L Nordén A Sjölund K Stenstam M Hepatic injury in adult coeliac disease Lancet 1977 ii 270 272 69880 10.1016/s0140-6736(77)90954-0

[B12] Germenis AE Yiannaki EE Zachou K Roka V Barbanis S Liaskos C Adam K Kapsoritakis AN Potamianos S Dalekos GN Prevalence and clinical significance of immunoglobulin A antibodies against tissue transglutaminase in patients with diverse chronic liver diseases Clin Diagn Lab Immunol 2005 12 941 948 16085912 10.1128/CDLI.12.8.941-948.2005 PMC1182196

[B13] Teml A Vogelsang H Re: Celiac sprue: another autoimmune syndrome associated with hepatitis C Am J Gastroenterol 2001 96 2522 2523 10.1111/j.1572-0241.2001.04080.x 11513217

[B14] Bardella M Marino R Meroni PL Celiac disease during IFN treatment Ann Intern Med 1999 131 157 158 10419441 10.7326/0003-4819-131-2-199907200-00024

[B15] Cammarota G Cuoco L Cianci R Pandolfi F Gasbarrini G Onset of coeliac disease during treatment with interferon for chronic hepatitis C Lancet 2000 356 1494 1495 10.1016/S0140-6736(00)02880-4 11081540

[B16] Bourlière M Oulés V Perrier H Mengotti C Onset of celiac disease and interferon treatment Lancet 2001 357 803 804 10.1016/S0140-6736(05)71230-7 11253998

[B17] Adinolfi LE Durante Mangoni E Andreana A Interferon and ribavirin treatment for chronic hepatitis C may activate celiac disease Am J Gastroenterol 2001 96 607 608 10.1111/j.1572-0241.2001.03574.x 11232725

[B18] Hernandez L Johnson TC Naiyer AJ Kryszak D Ciaccio EJ Min A Bodenheimer HC Jr Brown RS Jr Fasano A Green PH Chronic Hepatitis C Virus and Celiac Disease, is there an Association? Dig Dis Sci 2008 53 256 261 10.1007/s10620-007-9851-z 17549632

[B19] Chomczynski P Sacchi N Single-step method of RNA isolation by aci guanidium thiocyanate-phenol-chloroform extraction Anal Biochem 1987 162 156 159 2440339 10.1006/abio.1987.9999

[B20] Kwok S Higuchi R Avoiding false positive with PCR Nature 1989 339 237 238 10.1038/339237a0 2716852

[B21] Viazov S Zibert A Ramakrishnan K Widell A Cavicchini A Schreier E Roggendorf M Typing of hepatits C virus isolates by DNA enzyme immunoassay J Virol Meth 1994 48 81 92 10.1016/0166-0934(94)90091-4 7962263

[B22] Ishak K Baptista A Bianchi L Callea F De Groote J Gudat F Denk H Desmet V Korb G MacSween RNM Phillips MJ Portmann BG Poulsen H Scheuer PJ Schmid M Thaler H Histological grading and staging of chronic hepatitis J Hepatol 1995 22 696 699 10.1016/0168-8278(95)80226-6 7560864

[B23] Brunt EM Pathology of nonalcoholic fatty liver disease Nat Rev Gastroenterol Hepatol 2010 7 195 203 10.1038/nrgastro.2010.21 20195271

[B24] Ciclitira PJ King A Fraser J AGA technical review on celiac sprue Gastroenterology 2001 120 1526 1540 a. Watt, Switzerland 10.1053/gast.2001.24056 11313324

[B25] Guadagnino V Stroffolini T Rapicetta M Costantino A Kondili LA Menniti-Ippolito F Caroleo B Costa C Griffo G Loiacono L Pisani V Focà A Piazza M Prevalence, risk factors, and genotype distribution of hepatitis C virus infection in the general population. A community-based survey in southern Italy Hepatology 1997 26 1006 1011 10.1002/hep.510260431 9328327

[B26] Ferguson A Arranz E O’Mahony S Clinical and pathological spectrum of coeliac disease – active, silent, latent, potential Gut 1993 34 150 151 10.1136/gut.34.2.150 8432463 PMC1373959

[B27] Fattovich G Giustina G Favarato S Ruol A A survey of adverse events in 11,241 patients with chronic viral hepatitis treated with alpha interferon J Hepatol 1996 24 38 47 8834023 10.1016/s0168-8278(96)80184-x

[B28] Thevenot T Denis J Jouannaud V Monnet E Renou C Labadie H Abdelli N Nguyen-Khac E Dumouchel P Bresson-Hadni S Chousterman M Di Martino V Cadranel JF Coeliac disease in chronic hepatitis C: a French multicentre prospective study Aliment Pharmacol Ther 2007 26 1209 1216 10.1111/j.1365-2036.2007.03499.x 17944735

[B29] Ruggeri C La Masa AT Rudi S Squadrito G Di Pasquale G Maimone S Caccamo G Pellegrino S Raimondo G Magazzù G Celiac disease and non-organ-specific autoantibodies in patients with chronic hepatitis C virus infection Dig Dis Sci 2008 53 2151 2155 10.1007/s10620-007-0146-1 18231858

